# Activation of *p53*, inhibition of telomerase activity and induction of estrogen receptor beta are associated with the anti-growth effects of combination of ovarian hormones and retinoids in immortalized human mammary epithelial cells

**DOI:** 10.1186/1475-2867-5-6

**Published:** 2005-03-08

**Authors:** Jiahui Zhang, Yifan Tu, Sallie Smith-Schneider

**Affiliations:** 1 Pioneer Valley Life Sciences Institute, Baystate Medical Center, 3601 Main Street, Springfield, MA 01199, USA

## Abstract

**Background:**

A full-term pregnancy has been associated with reduced risk for developing breast cancer. In rodent models, the protective effect of pregnancy can be mimicked with a defined regimen of estrogen and progesterone combination (E/P). However, the effects of pregnancy levels of E/P in humans and their underlying mechanisms are not fully understood. In this report, we investigated the growth inhibitory effects of pregnancy levels of E/P and both natural and synthetic retinoids in an immortalized human mammary epithelial cell line, 76N TERT cell line.

**Results:**

We observed that cell growth was modestly inhibited by E/P, 9-cis-retinoic acid (9-cis RA) or all-trans-retinoic acid (ATRA), and strongly inhibited by N-(4-hydroxyphenyl) retinamide (HPR). The growth inhibitory effects of retinoids were further increased in the presence of E/P, suggesting their effects are additive. In addition, our results showed that both E/P and retinoid treatments resulted in increased *RARE *and *p53 *gene activity. We further demonstrated that p53 and p21 protein expression were induced following the E/P and retinoid treatments. Furthermore, we demonstrated that while the telomerase activity was moderately inhibited by E/P, 9-cis RA and ATRA, it was almost completely abolished by HPR treatment. These inhibitions on telomerase activity by retinoids were potentiated by co-treatment with E/P, and correlated well with their observed growth inhibitory effects. Finally, this study provides the first evidence that estrogen receptor beta is up-regulated in response to E/P and retinoid treatments.

**Conclusion:**

Taken together, our studies show that part of the anti-growth effects of E/P and retinoids is p53 dependent, and involve activation of *p53 *and subsequent induction of p21 expression. Inhibition of telomerase activity and up-regulation of estrogen receptor beta are also associated with the E/P- and retinoid-mediated growth inhibition. Our studies also demonstrate that the potency of retinoids on cell growth inhibition may be increased through combination of estrogen and progesterone treatment.

## Background

It is well documented that women who experience a full-term pregnancy early in their lives have a significantly reduced risk for developing breast cancer [1,2]. The mechanisms by which pregnancy affects maternal breast cancer incidences are not fully understood. Previous studies suggest that the protective effect of pregnancy can be mimicked with a defined regimen of estrogen and progesterone combination (E/P) in rodent models [3,4]. However, the effects of pregnancy levels of E/P in human and their underlying mechanisms have not been investigated.

Retinoids are a promising class of chemopreventive agents against breast cancer because of their antiproliferative and proapoptotic properties [5,6]. Retinoic acid receptors (RARs) and retinoid X receptors (RXRs) are nuclear transcription factors that modulate the biological effects of retinoids. Most retinoid forms, including 9-cis-retinoic acid (9-cis RA) and all-trans-retinoic acid (ATRA), activate RAR family members, whereas RXR family members are activated by 9-cis RA. N-(4-hydroxyphenyl) retinamide (HPR), a synthetic derivative of ATRA, may weakly interact with retinoid receptors [7].

The 76N TERT cells were derived from a reduction mammoplasty specimen [8,9]. They are normal human mammary epithelial cells immortalized by plasmids containing hTERT, the human catalytic subunit of the reverse transcriptase protein of telomerase [10]. hTERT-expressing normal cell clones have been shown to have an extended life span without any change in karyotype [11]. The 76N TERT cells in culture could continuously grow about 60 population doublings [8], and the level of p53 protein has been shown to remain consistent at early or late passages [9]. Unlike the tumor cell lines widely used in breast cancer researches such as MCF-7 and MDA-MD-231 cells, which have undergone several steps in tumorigenesis, the 76N TERT cell line represents a system that is immortal but does not yet have the capacity to form a tumor. Hence, it is potentially a better model to study the genetic changes, and to test the effects of carcinogenic or chemopreventive agents on the development of mammary tumors.

In this study, we investigated whether E/P induce growth inhibition in 76N TERT cells; and the molecular mechanisms by which E/P inhibited 76N TERT cell growth. For comparison purpose, the anti-growth effect of both natural and synthetic retinoids was examined in parallel in this immortalized mammary epithelial cell line. We also investigated whether we could increase the responsiveness of retinoids by using retinoids in combination with E/P. Our studies demonstrate that 1) inhibition of cell growth by E/P and retinoids could be partially mediated through a p53-dependent mechanism; 2) induction of p21 expression, inhibition of telomerase activity, or up-regulation of estrogen receptor beta (ERβ) by E/P and retinoids may contribute to their anti-growth effects; 3) combination of retinoids with E/P lead to increased inhibitory effects on cell growth.

## Results

### Expression of RARs, RXRs, estrogen receptors and progesterone receptors in 76N TERT cells

**Figure 1:**
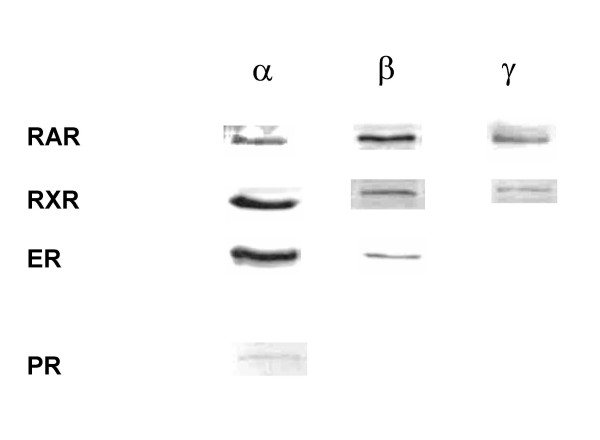
**Expression of RAR, RXR, ER and PR in 76N TERT cells. **Whole cell lysates were analyzed by using anti-RAR, RXR, ER, and PR antibodies as described under "Materials and Methods". Blots shown are representative of 2–3 experiments with similar results.

**Figure 6:**
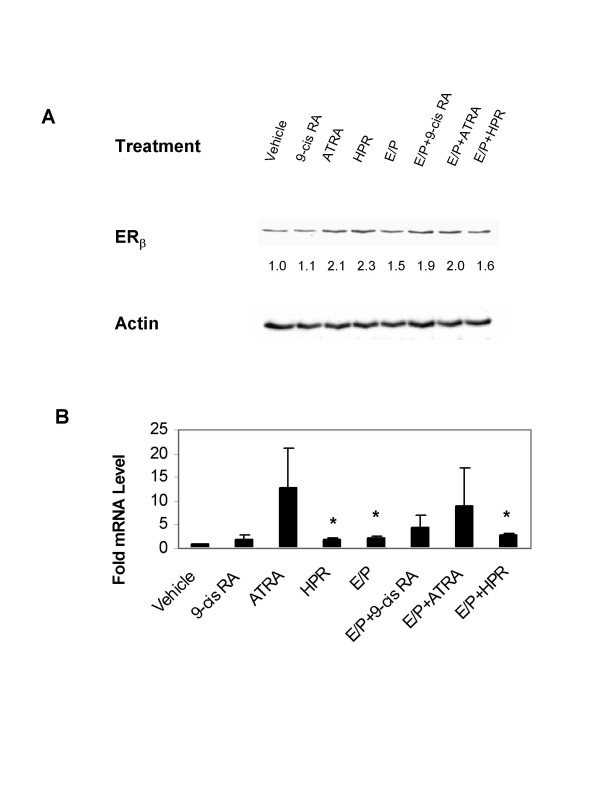
**Induction of ERβ expression by E/P and retinoids in 76N TERT cells**. Cells were treated with indicated retinoids with or without E/P for 72 hours. **A. **Western blot analysis of ERβ protein. Relative ERβ expression was normalized to actin protein and expressed as fold changes compared to vehicle treatment. Blot shown is representative of 3 experiments with similar results. **B. **Quantitative RT-PCR analysis of ERβ genes. Data are the means ± SE from 3 experiments. *, significant differences from their own controls.

As the ability of estrogen, progesterone and retinoids to influence cell proliferation is mediated by their respective receptors in most cases, we first examined the expression of these receptors in 76N TERT cells. Using Western blot analysis, the proteins of RAR (RARα, RARβ and RARγ), RXR (RXRα, RXRβ and RXRγ), and ER (ERα and ERβ) were observed as suggested by the manufactures (Figure 1). The antibody for the progesterone receptor (PR) detected a protein between 85 and 125 KDa (Figure 1). The existences of RARβ and ERβ in 76N TERT cells were further confirmed by quantitative RT-PCR at the mRNA level (RARβ data not shown, ERβ data see Figure 6B).

### Inhibition of 76N TERT cell growth by E/P and retinoids

**Figure 2:**
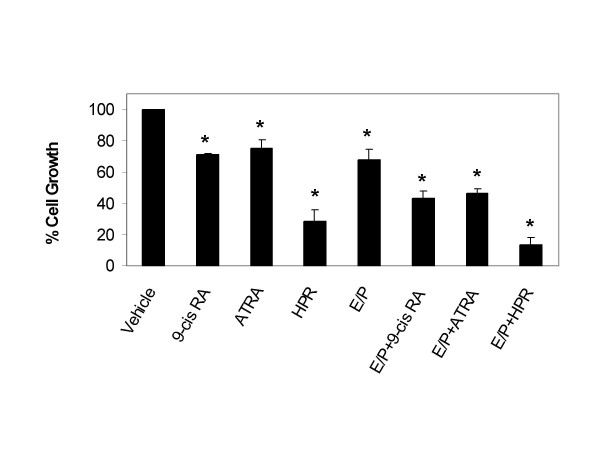
**Inhibition of 76N TERT cell growth by E/P and retinoids**. Cells were treated with indicated retinoids with or without E/P for 24 hours and then labelled with [^3^H]thymidine as described under "Materials and Methods". Data are the means ± SE from 3–4 experiments. *, significant differences from control.

We then tested the influence of E/P or retinoids on 76N TERT cell growth, using [^3^H]thymidine incorporation assay. The concentrations of E/P and retinoids used in the experiments were chosen based on previous studies and are clinically or physiologically relevant [3,4,12]. Treatment of cells with E/P or all three retinoids resulted in decreases in cell proliferation. As shown in Figure 2, treatment of 76N TERT cells with 1 μM 9-cis RA or 2 μM ATRA significantly decreased the cell growth by 28.8% and 24.5% respectively. In comparison, cells were more responsive to HPR. Treatment with 2 μM HPR exhibit a significant 71.4% decrease in cell growth relative to controls. Combination of 1 ng/ml of β-estradiol and 1 μg/ml of progesterone, a regime that mimics the protective effects of pregnancy, also resulted in a significant 32.2% decrease in cell proliferation. In the presence of E/P, the inhibitory effects on cell growth of 9-cis RA, ATRA and HPR were further increased to a respective 56.6%, 53.3% and 86.8%, indicating that the anti-proliferative effect may be additive between E/P and retinoids.

### Activation of *RARE *and *p53 *gene by E/P and retinoids

**Figure 3:**
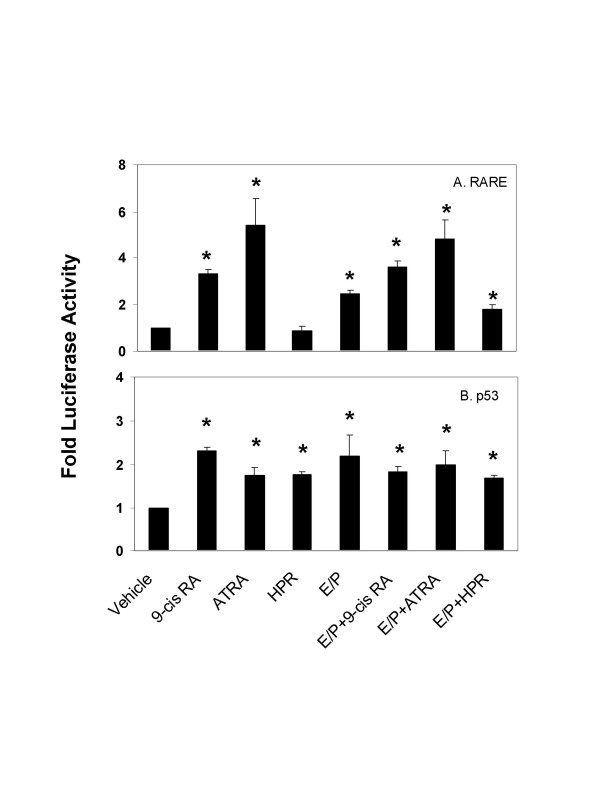
**Activation of *RARE *and *p53 *gene by E/P and retinoids in 76N TERT cells**. Cells were transiently transfected with the *RARE *(**A**) or *p53 *(**B**) reporter plasmid, and then treated with retinoids for 24 hours with or without E/P. Luciferase activity was measured and normalized as described under "Materials and Methods". Results are the means ± SE from 3 experiments. *, significant differences from control.

Many biological responses to retinoids are thought to be mediated through receptors by binding to retinoic acid response elements (*RARE*s) and regulation of transcriptional activity [5,6]. In addition, cross-talk between ER and RAR pathways has been previously reported [13,14]. Given this, a comparison of *RARE *gene activation in response to E/P and retinoids was carried out using cells transfected with a *RARE*-luciferase reporter gene construct. As shown in Figure 3A, 76N TERT cells exhibited a respective 3.3-, 5.4-, and 2.5-fold activation of *RARE *gene in response to 9-cis RA, ATRA or E/P alone. In contrast, HPR caused no significant change in luciferase activity relative to the control. In the presence of E/P, the effects of 9-cis RA and ATRA on *RARE *gene activation were essentially the same as without E/P.

Functional p53 provides a protective effect against tumor growth [3,15]. We next examined whether E/P and retinoids could enhance the transcriptional activity of *p53 *using 76N TERT cells transfected with a *p53*-responsive luciferase reporter gene construct. As shown in Figure 3B, a 2.3-, 1.8-, 1.8- and 2.2-fold induction of luciferase activity was observed by treatment of cells with 9-cis RA, ATRA, HPR and E/P respectively. Co-treatment with E/P and retinoids showed no additional activation of *p53 *gene as compared to their treatments alone.

### Induction of p53 and p21 protein expression by E/P and retinoids

**Figure 4:**
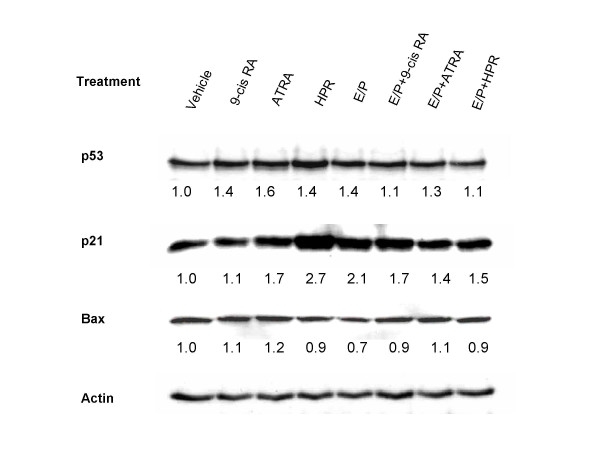
**Induction of p53 and p21 protein by E/P and retinoids in 76N TERT cells**. Cells were treated with indicated retinoids with or without E/P for 72 hours. Whole cell lysates were subjected to Western blot analysis using anti-p53, p21 and Bax antibodies. Relative expression of each protein was determined using the same membrane, and normalized to actin protein. Data are fold changes compared to vehicle treatment. Blot shown is representative of 4 experiments with similar results.

We then performed Western blot analysis to test whether increased *p53 *gene activity is paralleled by increased p53 protein expression. Treatment with 9-cis RA, ATRA, HPR or E/P alone slightly increased (about 1.5-fold) the expression of p53 protein. Consistent with the data on *p53 *gene activation, no additive effects between E/P and retinoids on induction of p53 protein were observed (Figure 4).

Activated p53 could induce the transcription of either p21 to cause growth arrest, or Bax to induce apoptosis [15]. We therefore investigated whether increased p53 protein expression can modulate the expression level of p21 or Bax proteins in 76N TERT cells. In Figure 4, exposure to retinoids or E/P alone did moderately increase the p21 protein level, with a 1.7-, 2.7- and 2.1-fold increase in p21 expression for ATRA, HPR and E/P respectively. The increases of p21 protein expression with combined E/P and retinoids were similar to that observed when E/P and retinoids were used alone. In addition, no significant effects by E/P or retinoids on Bax protein expression were observed in 76N TERT cells.

### Inhibition of telomerase activity by E/P and retinoids

**Figure 5:**
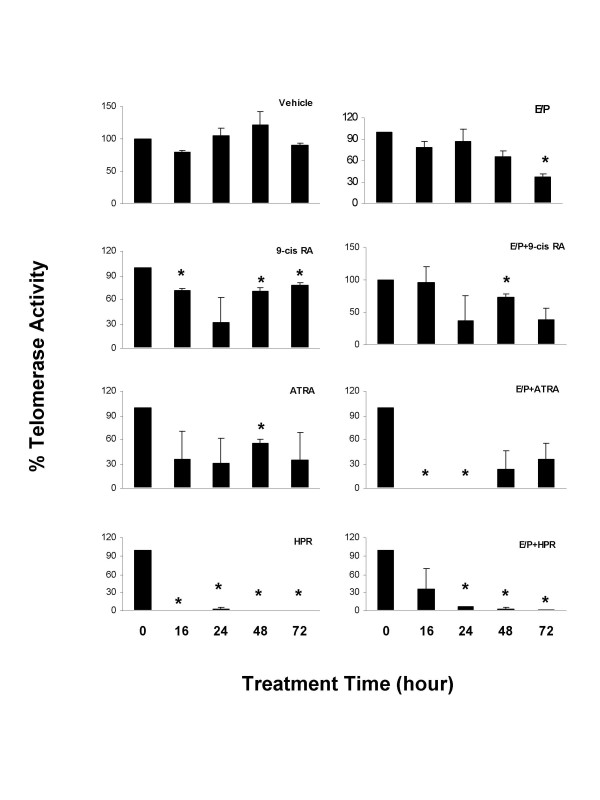
**Inhibition of the telomerase activity by E/P and retinoids in 76N TERT cells**. Cells were treated with indicated retinoids with or without E/P for the indicated time periods. Telomerase activity was determined as described under "Materials and Methods". Data are the means ± SE from 2–3 measurements. *, significant differences from their own controls.

Activation of telomerase is an early event in the development of breast cancers that may lead to cellular immortality, a critical and rate-limiting step in oncogenesis [16,17]. Activated *p53 *has been associated with regulation of the telomerase activity [18-20]. To evaluate the effects of E/P and retinoids on telomerase activity in 76N TERT cells, cells were treated for different time periods with E/P and retinoids, and the levels of telomerase activity were determined by a quantitative real-time PCR method. As shown in Figure 5, treatment with E/P decreased telomerase activity in a time-dependent manner with a 63.3% inhibition at 72 hours, whereas vehicle treatment had no effect at any time during the experiment. The maximum inhibition on telomerase activity was observed at 24 hours for both 9-cis RA and ATRA treatments, with a respective of 68.9% and 69.4% decrease. In comparison, the effects of HPR occurred more rapidly, with a complete inhibition at 16 hours, and persisted throughout the treatment. The inhibitory effects of 9-cis RA, ATRA or HPR and E/P seemed to be additive, as in the presence of E/P, 9-cis RA, ATRA or HPR showed increased inhibitions at various time points. These effects correlate well with their observed growth inhibitory effects in 76N TERT cells, suggesting that inhibition of telomerase activity by E/P and retinoids may contribute to their additive effects on inhibition of cell growth.

### Induction of ERβ expression by E/P and retinoids

There is growing evidence that ERβ could be an inhibitor of tumorigenesis of breast cancer [21-23]. We examined whether there were any changes in the expression of ERβ in response to E/P and retinoid treatment in 76N TERT cells. After treatment of cells with retinoids or E/P for 72 hours, the amount of ERβ protein was determined by Western blot analysis. As shown in Figure 6A, the normalized ERβ protein showed a respective 2.1-, 2.3-, and 1.5-fold increase in response to ATRA, HPR and E/P, as compared to the vehicle treatment.

We also carried out a quantitative analysis of ERβ mRNA expression in response to E/P and retinoid treatment in 76N TERT cells using a real-time PCR assay. As shown in Figure 6B, there was a 2.0-, 2.3- or 2.8-fold induction of ERβ mRNA levels in HPR, E/P or combination of HPR and E/P treated cells, respectively.

## Discussion

Similar to retinoids, a full-term pregnancy has been associated with beneficial effects on breast cancer risk over the long term [1,2]. The mechanisms by which pregnancy affects maternal breast cancer incidences are not fully understood. Studies have showed that higher concentrations of progesterone elicit a growth-inhibiting response from normal and cancerous breast cells [24,25], and are inversely related to breast cancer incidence [26]. In this study, we examined the ability of pregnancy levels of E/P and retinoids to affect the growth of the immortalized normal mammary epithelial cells. Our results demonstrated that three isoforms for RAR and RXR (α, β, γ), two isoforms for ER (α and β), and PR receptor proteins are expressed by 76N TERT cells. Treatment with 9-cis RA, ATRA, HPR or E/P inhibited 76N TERT cell proliferation and resulted in the activation of *p53 *gene, followed by increased expression of p53 protein and p21 protein, and inhibition of telomerase activity. Additionally, we first report here that the expression of ERβ is induced in response to E/P and retinoid treatment at both the transcriptional and translational levels. Importantly, we demonstrate that the inhibitory effects of retinoids on cell growth are more effective in the presence of E/P, and correlate well with their inhibitory effects on telomerase activity in 76N TERT cells.

Our data suggest that the growth inhibitory effects of E/P and retinoids may involve the activation of p53 pathway in 76N TERT cells. First, our results showed that both E/P and retinoid treatments lead to the increased *p53 *gene activity. Secondly, we demonstrated that the protein expression of p53 and p21 were increased following the treatment. It has been shown that p21 can inhibit cyclin A/cdk2 kinase activity and subsequently result in cell cycle arrest [27,28]. Our data is in line with the previous findings that in normal mammary epithelial cells, retinoids induce cell cycle arrest which is associated with an increase in p21 expression [29]; and that in both rats and mice, *p53 *is activated in response to E/P and this activation is sustained to induce p21 [3]. Thirdly, our data showed that treatment with E/P or retinoids decreased the telomerase activity in 76N TERT cells. Although a few reports suggest that telomerase activity appears to be independent of *p53 *expression or mutation [30,31], the majority of the evidence to date support the involvement of *p53 *in regulation of telomerase activity in mammary epithelial cells and breast cancer [18-20]. The molecular mechanisms of regulation of telomerase activity by *p53 *may involve down-regulation of hTERT transcription or the interaction of *p53 *with other transcription factors [19].

However, our data also suggest the possibility that inhibition of cell growth by E/P and retinoids may be independent of p53 pathway in 76N TERT cells. Our data show that the enhancing effects of retinoids on *p53 *gene activation or on the p53 and p21 protein expression were not further augmented by the addition of E/P, unlike their inhibitory effects on cell growth, indicating that other mechanisms besides the p53 pathway are likely to be involved. A p53-independent cell cycle arrest by retinoids has been previously suggested in a number of breast carcinoma cells [27,32]. The p53-independent inhibitory effects of retinoids on cell growth can be exerted through various mechanisms including regulation of other genes that play critical roles in cell cycle progression such as c-myc [18], inhibition of activator protein mediated transcription [33], or induction of caspase-independent cell death via calcium and calpain [34]. However, the mechanisms of E/P-mediated p53-independent cell growth inhibition are still unknown, and are currently under investigation using cell lines with different functional p53 systems.

Clearly, our data suggest that there are some overlaps between E/P- and retinoid-mediated growth inhibition in 76N TERT cells, considering that in response to E/P and retinoid treatments, same effectors such as *RARE *and *p53 *gene, p53 and p21 protein, and the telomerase activity were affected. Additionally, there also seems to be cross-talk between the E/P- and retinoid-mediated growth inhibition. Previous studies have suggested that there is cross-talk between ERβ and RAR pathways [13,14,35]. Here, we demonstrated that the *RARE *gene activity was increased in response to E/P treatment. Furthermore, for the first time, we showed that treatment of immortal cells with E/P or retinoids could induce the expression of ERβ, both at the mRNA and protein level. The expression of ERβ often is found to be decreased markedly in the early stages of mammary carcinogenesis [22]. Loss of ERβ expression has been suggested as one of the events leading to the development of breast cancer [36]. Our data may reveal another important mechanism by which E/P and retinoids produce their anticancer function, indicating ERβ may represent a possible therapeutic target in breast cancer prevention.

More importantly, our data show that the growth-inhibitory effects of retinoids were potentiated by co-treatment with E/P in 76N TERT cells. These observations indicate that different mechanisms may be involved in E/P- and retinoid-mediated inhibition of cell growth. Our results of their differential inhibitory effects on telomerase activity may provide some explanation for this. Although E/P and all three retinoids inhibited telomerase activity, the time courses of their actions were different. While retinoids produced their maximal inhibitory effects around 16 to 24 hours after treatment, E/P required 72 hours to reach its maximal inhibition, suggesting different mediators may be utilized to decrease telomerase activity by E/P and retinoids. ATRA and 9-cis RA have been previously reported to inhibit cell growth and decrease telomerase activity through down-regulation of the expression of hTERT telomerase gene [37]. On the other hand, progesterone treatment has been shown to down-regulate telomerase activity by modulation of cell cycle phases [38]. Previous studies have also provided evidence that the function of *p53 *in suppression of telomerase activity is separable from its cell cycle checkpoint function [20]. Therefore, it is likely that even though E/P and retinoids treatments both activate p53 pathway, they may use different mechanisms to inhibit telomerase activity. The different mediators involved in the inhibitory effects of E/P and retinoids on telomerase activity may contribute to their additive effects on inhibition of 76N TERT cell growth. The detailed mediator mechanisms down-stream of p53 and up-stream of telomerase activity for both E/P and retinoid pathways remain to be defined.

Several lines of evidence suggest that the mechanisms through which HPR regulates cell growth seem different than those by 9-cis RA and ATRA in 76N TERT cells. In the [^3^H]thymidine incorporation experiments, our results showed that whereas only 25–30% inhibition was observed for 9-cis RA and ATRA, 70% inhibition was reached by HPR. In addition, in the *RARE*-luciferase activity assay, 9-cis RA and ATRA induce about 3- to 5-fold activation on *RARE *gene activity. In contrast, HPR treatment resulted in no significant change. Finally, our data showed that 9-cis RA or ATRA treatment caused a moderate inhibitory effect on telomerase activity. In comparison, the telomerase activity is almost completely abolished by HPR treatment. The time courses of their inhibition of telomerase activity were different as well. While 9-cis RA and ATRA maximally inhibited the telomerase activity around 24 hours after treatment, HPR produced its maximal effect at 16 hours post-treatment. An obvious explanation for these different responses observed between 9-cis RA, ATRA and HPR is that these retinoids most likely possess different mechanisms for their actions. As suggested by numerous investigators, 9-cis RA and ATRA may function through the classical retinoid pathways involving the RARs and RXRs. On the other hand, in addition to activation the retinoid receptors [7a], HPR may also function through alternative pathways such as down-regulation of the IGF system [39], activation of TGF-beta [40], induction of genes which have antiproliferative activity [41], inhibition of aromatase activity and expression [42], and involvement of cellular signals such as reactive oxygen species [43] and the sphingolipid ceramide [44].

## Conclusion

In summary, our data demonstrate that 76N TERT cells express RAR, RXR, ER and PR, and represent a potential useful model to investigate the genetic changes, and the carcinogenic or chemopreventive effects of new agents on the development of mammary tumors. In addition, our data clearly suggest that part of the anti-growth effects of E/P is mediated through a p53-dependent pathway, as well as the involvement of the inhibition of telomerase activity and induction of ERβ. Comparing the E/P- and retinoid-mediated inhibitory effects on cell growth, there are overlaps, cross-talks and distinct effectors between these pathways. Furthermore, our studies suggest that retinoids may be more effective when combined with E/P to prevent breast cancer development. This increased potency and sensitized response to retinoids with combined E/P treatment might have several important clinical implications for anti-cancer agents that mimic E/P effects. Firstly, it might allow the currently used RA regimens to show improved response in cancer prevention. Secondly, it may be sufficient to overcome some acquired or intrinsic RA resistance in cancer cells. Finally, it may lower the required does of either classes of anticancer agents used, leading to less side effects or toxicity. Overall, our studies better the understandings of the common and the unique mechanisms by which E/P and retinoids regulate cell growth, and may help us to design or to improve the clinical applications of anti-cancer agents.

## Methods

### Chemicals

ATRA, 9-cis RA, HPR, β-estradiol and progesterone were all purchased from Sigma (St. Louis, MO, USA) and dissolved in ethanol. The final concentration was 2 μM for ATRA and HPR, 1 μM for 9-cis RA, 1 ng/ml for β-estradiol and 1 μg/ml for progesterone. These concentrations were chosen based on previous studies and are clinically or physiologically relevant [3,4,12].

### Culture of 76N TERT cells

Cell line was originally supplied and cultured as described by Band et al. [8]. The culture medium D-MEM/F-12, fetal bovine serum, penicillin, streptomycin, and gentamicin were from Gibco (Carlsbad, CA, USA). All the other cell culture reagents were from Sigma (St. Louis, MO, USA). Cells were grown in D-MEM/F-12 mixture (1:1, vol/vol) containing 15 mM HEPES buffer and 2.5 mM L-glutamine, supplemented with 1% fetal bovine serum, 12.5 ng/ml epidermal growth factor, 10 nM triiodothyronine, 50 μM freshly made ascorbic acid, 2 nM estradiol, 1 μg/ml insulin, 2.8 μM hydrocortisone, 0.1 mM ethanolamine, 0.1 mM phosphorylethanolamine, 10 μg/ml transferrin, 15 nM selenite, 1 ng/ml cholera toxin, 35 μg/ml bovine pituitary extract, 100 units/ml penicillin, 100 mg/ml streptomycin, and 20 μg/ml gentamicin. Cells were maintained in 95% humidified air plus 5% CO_2 _and sub-cultured weekly. All experiments were performed on cells with passage numbers from 6 to 15.

### [^3^H]Thymidine Incorporation Assay

Cells were seeded into 24-well plates at 5 × 10^4 ^cells per well and incubated at 37°C overnight. Cells were then treated in triplicates with indicated retinoids in the presence or absence of E/P for 24 hours. After labelling cells with 1 μCi/ml of [^3^H]thymidine (Amersham, Arlington Heights, IL, USA) for 24 hours, cells were harvested by washing with PBS and 10% TCA, solubilizing with the mixture of 0.1% SDS and 0.1N NaOH. Aliquots were taken for the quantification of radioactivity by the Tri-Carb 2900 TR Liquid Scintillation Analyzer (Perkin Elmer, Wellesley, MA, USA). Incorporation of [^3^H]thymidine was expressed as a fold change from vehicle control under the same conditions.

### Luciferase Reporter Assays

Cells were seeded in 24-well plates at 5 × 10^4 ^cells per well. Cells were transiently transfected with 0.5 μg of either a *RARE*-luciferase or a *p53*-luciferase plasmid along with 0.05 μg of pCMV-Renilla luciferase using the SuperFect transfection reagent (QIAGEN, Valencia, CA, USA), following the manufacturer's recommended procedure. Twenty-four hours after transfection, triplicate cultures were treated with retinoids for 24 h in the presence or absence of E/P. The cells were then washed and lysed. The luciferase activities were measured using the DUAL-luciferase Assay System (Promega, Madison, WI, USA), and normalized by pCMV-Renilla luciferase activity for each sample.

### Real-Time PCR Telomerase Activity Assay

Cells were lysed in CHAPS lysis buffer (Chemicon International, Temecula, CA, USA) and incubated at 4°C for 30 min. The lysate was then centrifuged at 12000 × *g *for 20 min at 4°C, and the supernatant was collected. The protein concentration was measured in each extract using the BCA protein Assay Reagent Kit (Pierce, Rockford, IL, USA). Telomerase activity was determined in duplicates by a quantitative real-time PCR telomerase detection Kit (Allied Biotech, Ijamsville, MD, USA) according to the manufacturer's protocol, using Mx4000 Multiplex Quantitative PCR System (Stratagene, La Jolla, CA, USA).

### Quantitative RT-PCR for ERβ gene analysis

Cells at subconfluence were treated with retinoids in the presence or absence of E/P for 72 hours. Total RNA was extracted with TRIZOL Reagent from Gibco (Carlsbad, CA, USA) according to the instructions of the manufacture. Single-stranded cDNA was made from 1 μg of total RNA with the Cells-to-cDNA kit (Ambion, Inc., Austin, TX, USA) at 42°C for 15 min. The primers for ERβ were 5'CGA TGC TTT GGT TTG GGT GAT 3' (forward) and 5'GCC CTC TTT GCT TTT ACT GTC 3' (reverse). The primers for GAPDH were 5'CCA TGG AGA AGG CTG GGG 3' (forward) and 5'CAA AGT TGT CAT GGA TGA CC 3' (reverse). All primers were from Integrated DNA Technologies, Inc. (Coralville, IA, USA). cDNA (1 μl) was amplified in duplicates in Mx4000 Multiplex Quantitative PCR System (Stratagene, La Jolla, CA, USA) by using Brilliant SYBR Green QPCR Master Mix from Stratagene (La Jolla, CA, USA). The reaction was carried out at 95°C for 10 min to denature, 40 cycles of 95°C for 30 sec, 55°C for 60 sec, 72°C for 60 sec. ERβ gene was quantified and normalized with external standard GAPDH.

### Western blot analysis

Cells were treated with retinoids in the presence or absence of E/P for 72 hours. Cell lysates were obtained by incubating cells for 30 minutes at 4°C in a buffer containing 50 mM Tris-HCl (pH 7.4), 150 mM NaCl, 0.5% NP40, 100 mM NaF, 10 mM MgCl_2_, and protease inhibitor cocktail (Sigma, St. Louis, MO, USA), followed by centrifuged at 12,000 rpm for 20 min. Protein content was determined using the BCA Protein Assay Reagent Kit (Pierce, Rockford, IL, USA). Cell lysates (~30 μg protein) were separated on 10% polyacrylamide gels in the presence of 0.1% SDS with prestained low-range molecular-weight standards (Biorad, Richmond, CA, USA). After transfer, the membranes were blocked and then probed with antibodies against interested proteins as suggested by manufactures, followed by incubation with a peroxidase-conjugated secondary antibody. Immunoreactive bands were developed with an ECL reagent from Amersham (Arlington Heights, Il, USA). All blots were probed with anti-actin to normalize for loading differences. Quantification of gels was carried out using ImageJ software (NIH, Bethesda, Maryland, USA).

The p53 (DO-1), p21 (F-5) and RARγ (G-1) mouse monoclonal antibodies, and RXRα (D-20) polyclonal antibody were from Santa Cruz Biotechnology Inc (Santa Cruz, CA, USA). Anti-ERβ (Ab-2), ER (Ab-1), PR (Ab-1), RARα and RARβ mouse monoclonal antibodies, and Bax (Ab-1) polyclonal antibody were from Calbiochem (San Diego, CA, USA). Monoclonal anti-actin, RXRβ and RXRγ were from Sigma (St Louis, MO, USA).

### Statistical Analysis

Statistical differences were analyzed by T-test. Levels of statistical significance were set at p < 0.05.

## List of abbreviations

9-cis RA: 9-cis-retinoic acid; ATRA: all-trans-retinoic acid; E/P: ?β-estradiol and progesterone; ERβ: estrogen receptor beta; HPR: N-(4-hydroxyphenyl) retinamide; PR: progesterone receptors; RARs: retinoic acid receptors; RXRs: retinoid X receptors.

## Competing interests

The author(s) declare that they have no competing interests.

## Authors' contributions

JZ designed and carried out most of the assays and drafted the manuscript. YT carried out the real-time PCR experiments. SS conceived of the study and participated in its coordination. All authors read and approved the final manuscript.
